# Composition and Localization of Lipids in *Penaeus merguiensis* Ovaries during the Ovarian Maturation Cycle as Revealed by Imaging Mass Spectrometry

**DOI:** 10.1371/journal.pone.0033154

**Published:** 2012-03-14

**Authors:** Piyachat Chansela, Naoko Goto-Inoue, Nobuhiro Zaima, Takahiro Hayasaka, Morakot Sroyraya, Napamanee Kornthong, Attakorn Engsusophon, Montakan Tamtin, Chatchawalee Chaisri, Prasert Sobhon, Mitsutoshi Setou

**Affiliations:** 1 Faculty of Science, Department of Anatomy, Mahidol University, Bangkok, Thailand; 2 Department of Cell Biology and Anatomy, School of Medicine, Hamamatsu University, Hamamatsu, Shizuoka, Japan; 3 Graduate School of Health Promotion Sciences, Tokyo Metropolitan University, Hachioji, Tokyo, Japan; 4 Department of Applied Biological Chemistry, Kinki University, Nara, Japan; 5 Department of Fisheries, Coastal Aquatic Feed Research Institute, Coastal Fisheries Research and Development Bureau, Petchaburi, Thailand; Konkuk University, Republic of Korea

## Abstract

Ovary maturation, oocyte differentiation, and embryonic development in shrimp are highly dependent on nutritional lipids taken up by female broodstocks. These lipids are important as energy sources as well as for cell signaling. In this study, we report on the compositions of major lipids, i.e. phosphatidylcholines (PCs), triacylglycerols (TAGs), and fatty acids (FAs), in the ovaries of the banana shrimp, *Penaeus merguiensis*, during ovarian maturation. Thin-layer chromatography analysis showed that the total PC and TAG signal intensities increased during ovarian maturation. Further, by using gas chromatography, we found that (1) FAs 14∶0, 16∶1, 18∶1, 18∶2, 20∶1, and 22∶6 proportionally increased as ovarian development progressed to more mature stages; (2) FAs 16∶0, 18∶0, 20∶4, and 20∶5 proportionally decreased; and (3) FAs 15∶0, 17∶0, and 20∶2 remained unchanged. By using imaging mass spectrometry, we found that PC 16∶0/16∶1 and TAG 18∶1/18∶2/22∶6 were detected in oocytes stages 1 and 2. PCs 16∶1/20∶4, 16∶0/22∶6, 18∶3/22∶6, 18∶1/22∶6, 20∶5/22∶6, and 22∶6/22∶6 and TAGs 16∶0/16∶1/18∶3, 16∶0/18∶1/18∶3, 16∶0/18∶1/18∶1, and 16∶0/18∶2/22∶6 were present in all stages of oocytes. In contrast, the PC- and TAG-associated FAs 20∶4, 20∶5, and 22∶6 showed high signal intensities in stage 3 and 4 oocytes. These FAs may act as nutrition sources as well as signaling molecules for developing embryos and the hatching process. Knowledge of lipid compositions and localization could be helpful for formulating the diet for female broodstocks to promote fecundity and larval production.

## Introduction

The banana shrimp, *Penaeus merguiensis*, is one of the most important commercial shrimps in the Indo-Pacific region [1]. In Thailand, more than 90% of the shrimp production is cultured, and the most popular shrimp species which have been selected for cultured are *Penaeus monodon* and *Litopenaeus vannamei*. The former is considered to have superior meat quality and commands higher price, while the latter is an imported species having lower quality meat. However, the large scale and high density of *P. monodon* farming caused increases of disease outbreaks. An alternative indigenous species of equal quality is the banana shrimp, *P. merguiensis*, which is also highly favored by consumers [1], [2]. This species has a large natural population that can provide sufficient broodstocks; however, to have any commercially viable aquaculture the broodstocks must be domesticated and provided with suitable feeds which must be formulated based on the lipid compositions of the developing and ripe gonads. Recent attempts to culture this species employed practical procedures based entirely on the knowledge of *P. monodon*. Since the details of gonadal lipids profiles of this species could be different from other marine species, to improve the production of *P. merguiensis*, especially female broodstocks, information on ovarian lipid composition is important as it could be used for formulating a suitable feed to be used as a convenient diet for the broodstock. During ovarian maturation, lipids are one of the essential molecules taken up by female broodstocks and stored in the oocytes to nourish subsequent embryonic development. The composition of lipids in the ovaries of several shrimp species have been studied, including *Penaeus semisulcatus* [3], *Chorismus antarcticus* [4], *L. vannamei* [5], *Pandalus montagui* [6], *Penaeus esculentus* [7], and *Macrobrachium rosenbergii* [8]. In general, dietary lipids are the essential sources for fatty acid, phospholipid, sterol, and carotenoid syntheses [9]. Most of the lipids are synthesized and stored in the hepatopancreas before being transported to the ovary to be incorporated into oocytes at various stages during the ovarian cycle [3]. However, the ability to synthesize certain essential lipids, including polyunsaturated fatty acids (PUFAs), is limited in shrimps, so that these lipids must be acquired from the diet [10]. These lipids have effects on growth and reproductive success in female shrimps through stimulating ovarian maturation and oocyte differentiation [10], [11]. In addition, phosphatidylcholine (PC) and triacylglycerol (TAG) are reported to be predominant lipids in shrimp ovaries and are required for embryogenesis, improvement in hatching, and nauplii production [12]. A number of studies on fatty acid (FA), PC, and TAG compositions in the ovaries of several shrimp species has been reported [5], [7], [13], [14]; however, no similar studies have been conducted in *P. merguiensis*. In this study, we focused on the compositions of 3 kinds of lipids,i.e., PCs, TAGs, and total FAs including PUFAs, particularly linoleic acid (18∶2), linolenic acid (18∶3), arachidonic acid (ARA, 20∶4), eicosapentaenoic acid (EPA, 20∶5), and docosahexaenoic acid (DHA, 22∶6). Moreover, we examined the localization of PCs, TAGs, and associated FAs in the *P. merguiensis* ovaries using imaging mass spectrometry (IMS). IMS is a technique that is useful for detecting the distribution of analytes directly on the tissue surface. The analytes that can be detected from this technique include pharmaceuticals, lipids, peptides, proteins, metabolites, and polymers [15], [16], [17], [18], [19], [20]. Since small metabolites are highly abundant in living organisms and easily detected by mass spectrometry, especially phospholipids and glycerolipids [21], [22], the IMS technique is a powerful tool for detecting these types of molecules and can performed without the requirement for labels [23], [24]. According to this technique, after the laser irradiation of a tissue surface, analyte maps were constructed from the several hundred types of molecules that were detected [25]. Lipid accumulation is an important process for promoting successive hatching and embryo development; however, this technique has not been used to examine the distribution of individual lipids in a penaeid shrimp. The distribution of lipids, as determined in this study using the IMS technique, provided information on the changes in lipid accumulation during ovarian maturation. This information could be used to formulate the diet of the female broodstock of this shrimp, thereby help to promote optimal ovarian development and oocyte differentiation, and to improve embryo development and egg production and quality.

## Results

### The histology of 4 ovarian stages during the ovarian cycle

**Figure 1:**
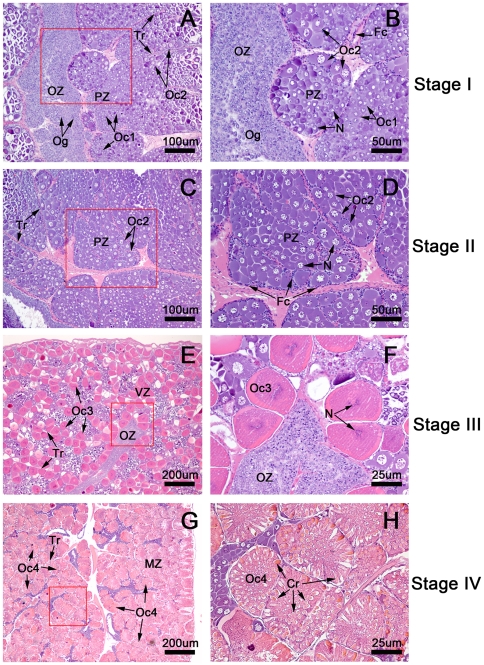
Hematoxylin-and eosin (H&E)-stained paraffin-embedded sections of ovaries at various stages. The ovaries are composed of lobules separated by connective tissue septae called trabeculae (Tr), with each lobule containing a variety of oocytes surrounded by follicular cells. (A,B) In the stage I ovary (the spent stage), the germ cells present are the oogonia (Og) in the oogenic zone (Oz) and stage 1 oocytes (Oc1) and stage 2 oocytes (Oc2) in the previtellogenic zone (PZ). (C,D) In the stage II ovary (the proliferative stage), the predominant cells are the stage 2 oocytes (Oc2) located in the previtellogenic zone (Pz), while Og and Oc1 are present to a lesser degree. (E,F) In stage III ovaries (the premature stage), the majority of oocytes at stage 3 oocytes (Oc 3) are located in the vitellogenic zone (VZ). Oc3 increase in size during maturation and contain increasing amounts of lipid droplets. The cytoplasm of these oocytes stains pink due to increased eosinophilia. (G,H) In the stage IV ovary (the mature stage), the mature oocytes (Oc4) are the largest and most abundant cells. Oc4 are located in the mature zone (MZ), which occupies almost the entirety of each lobule. The mature oocytes are identifiable by the highly eosinophilic cytoplasm, which is stained a deep pink by the eosin. The cytoplasm of these oocytes contains cortical rods; the oocytes are surrounded by follicular cells (Fc).

To obtain the ovarian histology, hematoxylin- and eosin (H&E)-stained ovarian sections from 4 ovarian stages were examined and photographed by a Nikon light microscope equipped with a digital camera E600. Each ovary was observed to be separated into lobules by connective tissue trabeculae (Tr) (Fig. 1A, C, E, G). In addition, each lobule was in contact with an oogenetic zone (OZ), which is an epithelial component lining the central ovarian cavity and containing the oogonia (Og) (Fig. 1A, C). Depending on the stage, each lobule might contain variable areas of a previtellogenic zone (PZ) (Fig. 1C, D), a vitellogenic zone (VZ) (Fig. 1E, F), and a mature zone (MZ) (Fig. 1G, H). In the stage I (the spent stage) (Fig. 1A, B), the OZ was quite large, and each lobule contained a small PZ area with stage 1 oocytes (Oc1) as the predominant germ cells and some stage 2 oocytes (Oc2). The Og and Oc1 diameters were approximately 10–14 and14–21 µm, respectively. Hematoxylin stained the Og cytoplasm blue and the Oc1 a deeper blue. In the stage II ovary (the proliferative stage) (Fig. 1C, D), each lobule contained mostly the PZ, and the predominant germ cells were Oc2. These oocytes increased in size (diameter ranging between 25–40 µm) over the span of the PZ; the cytoplasm stained light blue, and the chromatin was less densely packed in the nucleus when compared to that of Og and Oc1. In the stage III ovary (the premature stage) (Fig. 1E, F), each lobule primarily contained the VZ. The majority of cells were stage 3 oocytes (Oc3) that increased in diameter, ranging between 45–77 µm. The cytoplasm stained purple pink because of increased eosinophilia; an increased accumulation of lipid droplets was also evident. The nuclei could not be clearly observed at this stage, owing to the beginning of germinal vesicle break down. In the final stage or stage IV ovary (Fig. 1G, H), also known as the mature stage, each lobule contained mostly the MZ. The most abundant germ cells were stage 4 (Oc4) or mature oocytes. The cytoplasm stained a deep pink because of very high eosinophilia; the nuclei could not be observed in these oocytes due to complete breakdown of the germinal vesicle. The most prominent feature unique to Oc4 was the presence of cortical rods (Cr) that were arranged radially throughout the cytoplasm. The Oc4 were the largest oocytes in the ovaries, with diameters ranging between 115–197 µm (Fig. 1G, H), and were surrounded by follicular cells, which are the supporting cells in the ovary.

### Identification of PCs and TGs by thin-layer chromatography

**Figure 2:**
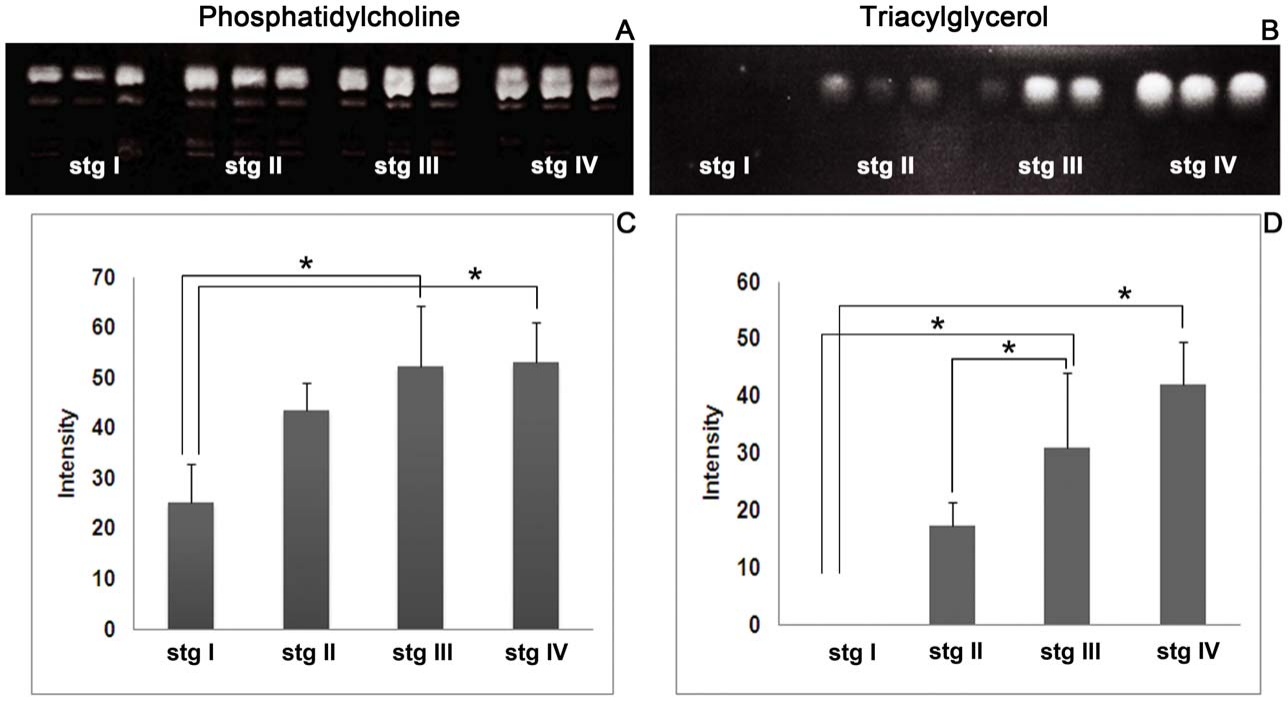
TLC-blots of lipids extracted from ovaries at various stages. (A) Phosphatidylcholines (PCs) separated on TLC-blot membranes, which show that the PC band increases in prominence from ovarian stages I to III and remains constant from ovarian stages III to IV. (C) The histograms of the signal intensities from TLC-blots confirm a similar trend in the changing levels of PC. (B) Triacylglycerides (TAGs) separated on TLC-blot membranes show that the TAGs are not detectable at stage I but dramatically increased from stages II–IV. (D)This pattern was also confirmed by histograms of TAG signal intensities. The significant differences of the results among the 4 stages were tested by Scheffe's method. The bars show standard deviations, and the asterisks indicate significant differences between stages (*p*<0.05).

Extracted lipids were separated by thin layer chromatography (TLC) to estimate the changing amounts of PCs and TAGs during the ovarian cycle. Figure 2 shows that the intensity of the PC band (Fig. 2A) dramatically increased from stage I to stage IV, whereas the TAG band (Fig. 2B) was absent in the stage I ovary, became increasingly prominent in stages II and III, and reached a maximum in stage IV. We validated that these bands were real PCs and TAGs by using TLC-Blot-MALDI MS (data not shown). Figure 2C and 2D show histograms of the calculated signal intensities of the PCs and TAGs. The results indicated that PC levels increased from stage I to stage III ovaries and remained constant from stage III to IV. On the other hand, during stage I, TAGs were barely detectable, but dramatically increased from stage II to IV.

### Identification of fatty acid (FA) compositions by GC-MS

**Figure 3:**
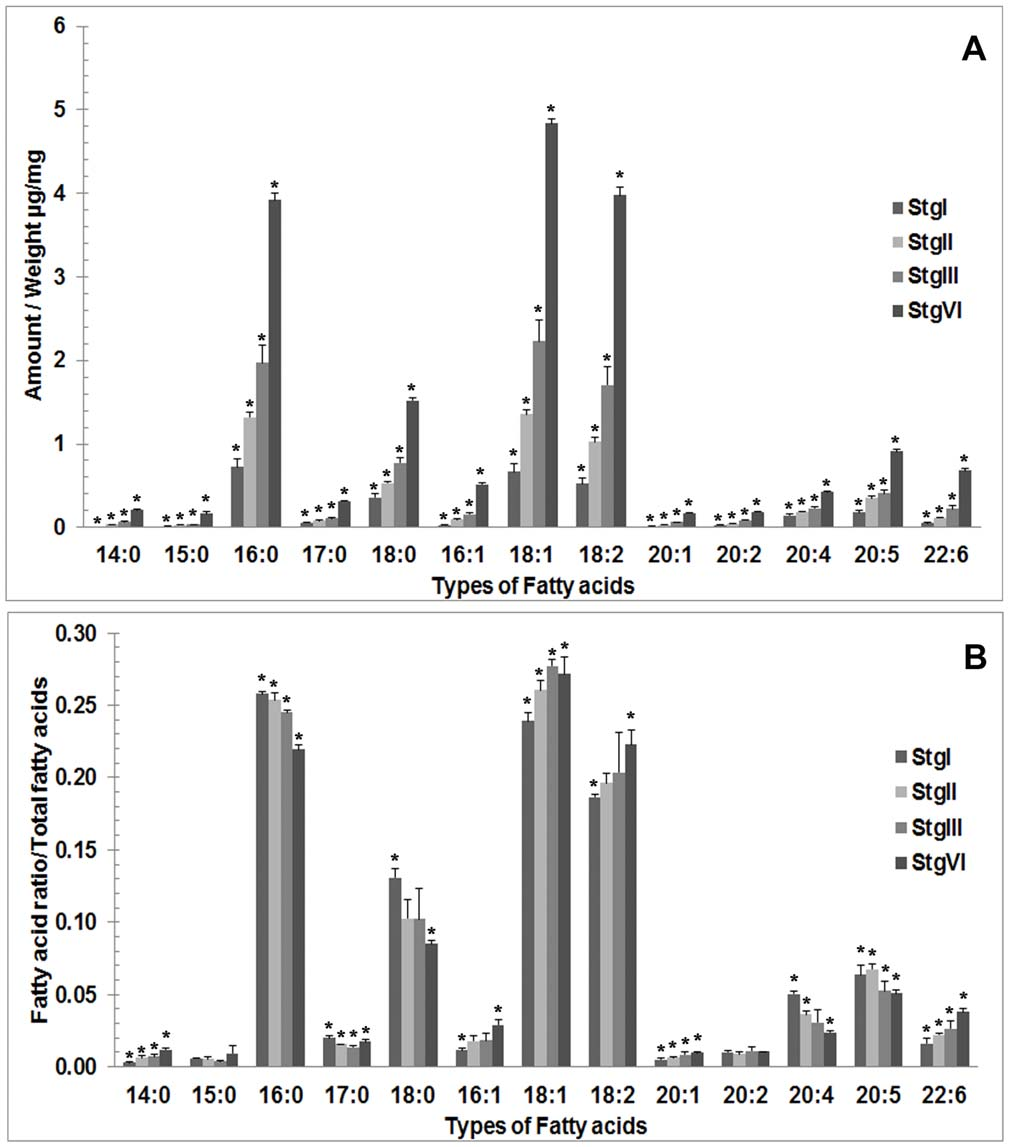
Histograms showing the average amount of each fatty acid (FA) per ovarian weight (µg/mg) (A) and the ratios between each FA per total amount of FAs (B) as detected in each ovarian stage. The average amounts of all the FAs tended to increase during ovarian development. However, the ratios between each FA per total amount of FA show 3 different patterns during the course of ovarian development. The ratios decreased for FAs 16∶0, 18∶0, 20∶4, and 20∶5; increased for FAs 14∶0, 16∶1, 18∶2, 18∶1, 20∶1, and 22∶6; and remained unchanged for FAs 15∶0, 17∶0 and 20∶2. Significant differences among the 4 stages for each FA were tested by Scheffe's method. The bars show standard deviations, and asterisks show significant difference for each fatty acid. (*p*<0.05).

We found that FAs in the *P. merguiensis* ovary were composed of both saturated and unsaturated FAs. The saturated FAs included tetradecanoic acid (14∶0), pentadecanoic acid (15∶0), hexadecenoic acid (16∶0), heptadecanoic acid (17∶0), and octadecanoic acid (18∶0), while the unsaturated FAs included hexadecenoic acid (16∶1), octadecenoic acid (18∶1), octadecadienoic acid (18∶2), eicosenoic acid (20∶1), eicosadienoic acid (20∶2), ARA (20∶4), EPA (20∶5), and DHA (22∶6). The amount of each FA per ovarian weight (µg/mg) was calculated by comparing it with the internal standard, eicosanoic acid (20∶0). Figure 3A shows histograms of the average amounts of FAs in each stage of the ovarian cycle. The following FAs were present, from the highest to lowest amount, in the ovaries: 18∶1, 16∶0, 18∶2, 18∶0, 20∶5, 20∶4, 22∶6, 16∶1, 17∶0, 20∶2, 20∶1, 14∶0, and 15∶0. We found that the amount of each FA significantly increased from stages I to IV. Based on the relative amounts in all 4 stages, FAs in the ovary can be classified into 4 groups, i.e., FAs showing the highest amount (16∶0,18∶1,18∶2), a moderate amount (18∶0), a low amount (17∶0, 16∶1, 20∶4, 20∶5, 22∶6), and the lowest amount (14∶0, 15∶0, 20∶1, 20∶2). The average amounts of FAs at the various ovarian stages are also shown in Table 1. To estimate the FA accumulation rates, the ratio of each FA to the total FAs was calculated (Fig. 3B). From these data, the FAs 14∶0, 16∶1, 18∶1, 18∶2, 20∶1, and 22∶6 were found to proportionally increase as ovarian development progressed to more mature stages. In contrast, the FAs 16∶0, 18∶0, 20∶4, and 20∶5 proportionally decreased. Finally, the FAs 15∶0, 17∶0, and 20∶2 were maintained at the same level throughout ovarian maturation. Changes in the amounts of FAs 14∶0, 16∶0, 17∶0, 18∶0, 16∶1, 18∶1, 18∶2, 20∶1, 20∶4, 20∶5 and 22∶6 were found to be statistically significant.

**Table 1:** The average concentrations of various fatty acids per ovarian weight in each ovarian stage as measured in GC-MS by comparing them with the internal standard (20∶0).

Fatty acid	Stage I (µg/mg) Mean ± SD	Stage II (µg/mg) Mean ± SD	Stage III (µg/mg) Mean ± SD	Stage IV (µg/mg) Mean ± SD
14∶0	0.008±0.001	0.035±0.003	0.062±0.009	0.209±0.005
15∶0	0.016±0.002	0.029±0.002	0.033±0.004	0.165±0.023
16∶0	0.725±0.102	1.319±0.056	1.968±0.215	3.917±0.084
17∶0	0.055±0.007	0.081±0.003	0.108±0.011	0.315±0.010
18∶0	0.361±0.048	0.530±0.019	0.775±0.059	1.520±0.032
16∶1	0.033±0.005	0.095±0.009	0.159±0.024	0.52 0±0.022
18∶1	0.666±0.091	1.355±0.061	2.232±0.247	4.838±0.052
18∶2	0.525±0.074	1.027±0.057	1.704±0.226	3.974±0.104
20∶1	0.015±0.002	0.033±0.003	0.063±0.005	0.172±0.004
20∶2	0.028±0.004	0.047±0.004	0.084±0.006	0.186±0.004
20∶4	0.142±0.020	0.186±0.005	0.227±0.014	0.425±0.006
20∶5	0.182±0.026	0.354±0.020	0.407±0.036	0.908±0.023

### Localization of lipids by imaging mass spectrometry

**Figure 4:**
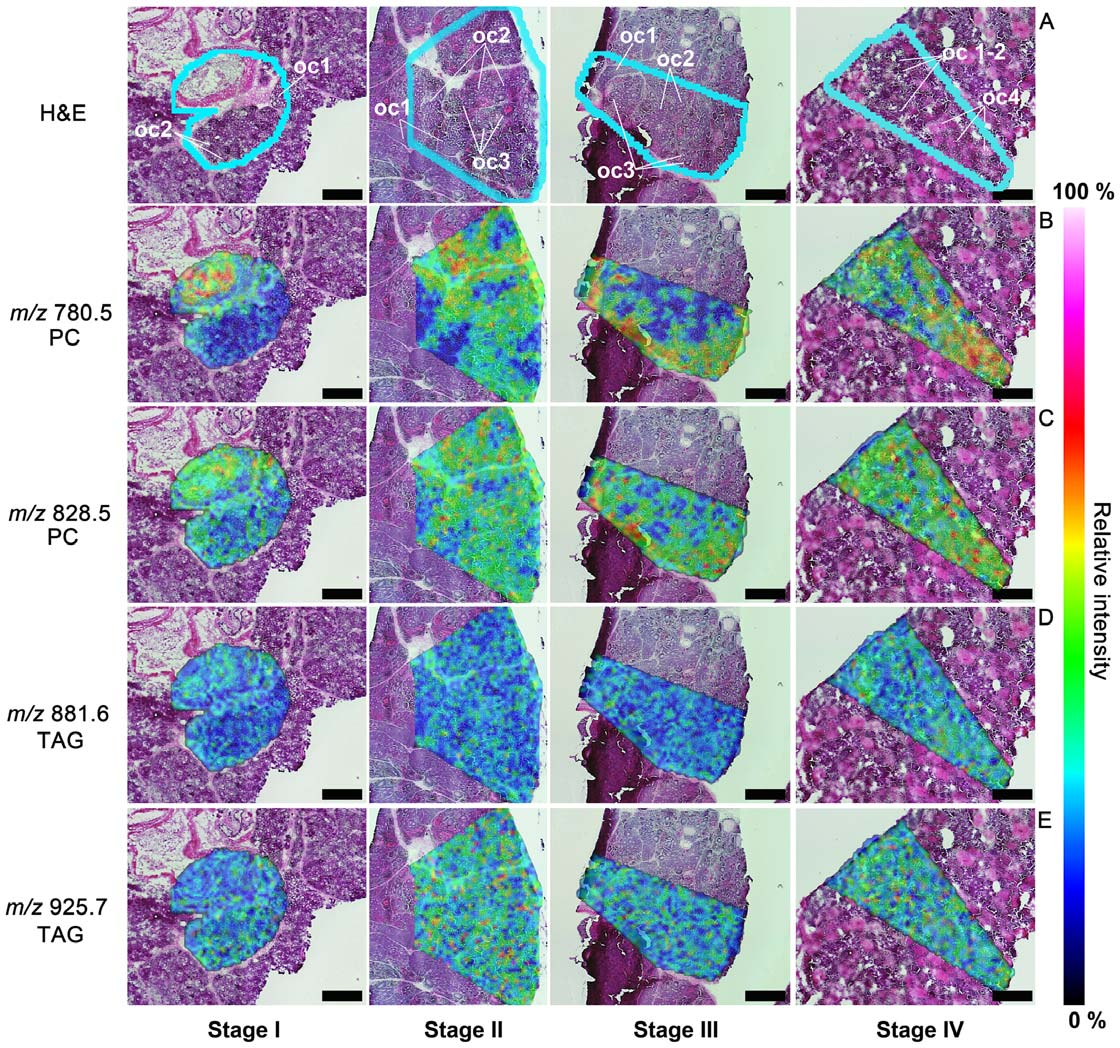
Imaging mass spectrometry (IMS) showing the distribution of PCs and TAGs on ovarian tissue sections. After IMS analysis, the tissue sections were further stained with H&E; the regions analyzed (blue lines) are shown on the H&E-stained sections (A). The observed signals from the IMS analysis were merged with the H&E images. These IMS images exhibit PC molecules at *m/z* 780.5 (B) and 828.5 (C) and TAG molecules at *m/z* 881.6 (D) and 925.7 (E). The PC molecule at *m/z* 780.5 showed intense distribution in late stage oocytes (Oc3 and Oc4) (B), whereas the molecule at *m/z* 828.5 showed distribution in all oocyte stages, with the most intense signal occurring in Oc3 and Oc4. For the TAGs, the molecules at *m/z* 881.6 and 925.7 were found distributed with similar intensity in all oocyte stages. The distribution of the other observed molecules are shown in Table 2 (the PCs) and 3 (the TAGs). The black scale bars represent 400 µm.

In this study, we observed specific PC and TAG signals that indicated their distributions in 3 different patterns, i.e. lipids localized mainly in Oc1,2 or Oc3,4 or present in all oocyte stages (Tables 2, 3). The first group of lipids detected in Oc1,2 comprised a PC at *m/z* 756.5 and a TAG at *m/z* 951.7. The second group detected with high signal intensities in Oc3,4 comprised PCs at *m/z* 780.5, 782.5, 804.5, 806.5, 808.5, 820.5, 826.5, 830.5, 832.5, 844.5, 852.5, and 870.5 (Table 2) and TAGs at *m/z* 823.6, 825.7, 851.7, 853.7, 877.7, and 879.7 (Table 3). The last group were lipids present in all oocyte stages and comprised PCs at *m/z* 802.5 828.5, 850.5, 854.5, 874.5, and 900.5 (Table 2) and TAGs at *m/z* 827.6, 855.6, 881.6, and 925.7 (Table 3). In this group, the signal intensities were found to be higher in Oc3 and Oc4 than in Oc1 and Oc2. Figure 4 shows the merged H&E-stained (Fig. 4A) and IMS images (Fig. 4B–E). The light blue lines indicate the regions where the IMS analyses were performed. The detected signals from PC molecules at *m/z* 780.5 (Fig. 4B) and 828.5 (Fig. 4C) and those from TAGs at *m/z* 881.6 (Fig. 4D) and *m/z* 925.7 (Fig. 4E) were selected to illustrate the distribution patterns. In Figure 4B, the signal of the molecule at *m/z* 780.5 was detected in Oc3,4 and dramatically increased in these cells during ovarian maturation. In contrast, the molecule at *m/z* 828.5 was distributed in all oocyte stages, with an especially high signal intensity detected in Oc3,4. For TAG, the molecules at *m/z* 881.6 and 925.7 were distributed in all oocyte stages, and the signal intensity slightly increased from ovarian stages I to IV. We have also provided the IMS images of other lipid molecules in Figures S1 and S2.

**Table 2:** Molecular phosphatidylcholine (PC) species and their distribution in shrimp ovaries.

*m/z*	Molecular species	adduct	Distribution
756.5	16∶0/16∶1	Na	Oocyte stages 1–2
780.5	16∶0/18∶2	Na	Oocyte stages 3–4
782.5	16∶0/18∶1	Na	Oocyte stages 3–4
802.5	16∶1/20∶4	Na	All stages
804.5	18∶2/18∶2	Na	Oocyte stages 3–4
806.5	18∶1/18∶2	Na	Oocyte stages 3–4
808.5	18∶0/18∶2	Na	Oocyte stages 3–4
820.5	16∶0/20∶4	K	Oocyte stages 3–4
826.5	18∶2/20∶5	Na	Oocyte stages 3–4
828.5	16∶0/22∶6	Na	All stages
830.5	18∶1/20∶4	Na	Oocyte stages 3–4
832.5	18∶0/20∶4	Na	Oocyte stages 3–4
844.5	16∶0/22∶6	Na	Oocyte stages 3–4
850.5	18∶3/22∶6	K	All stages
852.5	18∶2/22∶6	Na	Oocyte stages 3–4
854.5	18∶1/22∶6	Na	All stages
870.5	18∶1/22∶6	K	Oocyte stages 3–4
874.5	20∶5/22∶6	Na	All stages
900.5	22∶6/22∶6	Na	All stages

All PC species were verified by MS/MS analysis; however, complete fragment ion information was difficult to obtain for some PC molecules. Therefore, lipid molecules for which only partial fragment ions could be obtained were identified by an online database search (http://www.hmdb.ca/labm/jsp/mlims/MSDbParent.jsp) and Simlipid software version 1.0. The underlined molecules were identified from MS/MS analysis and had complete fragment ion spectra.

**Table 3:** Molecular species of TAGs and their distribution in the ovaries.

*m/z*	Molecular species	adduct	Distribution
823.6	16∶1/16∶1/16∶1	Na	Oocyte stages 3–4
825.7	16∶0/16∶1/16∶1	Na	Oocyte stages 3–4
827.6	16∶0/16∶1/18∶3	H	All stages
851.7	16∶0/16∶1/20∶5	H	Oocyte stages 3–4
853.7	16∶0/16∶0/20∶5	H	Oocyte stages 3–4
855.6	16∶0/18∶1/18∶3	H	All stages
877.7	16∶0/16∶0/20∶4	Na	Oocyte stages 3–4
879.7	18∶1/16∶0/20∶5	Na	Oocyte stages 3–4
881.6	16∶0/18∶1/18∶1	Na	All stages
925.7	16∶0/18∶2/22∶6	Na	All stages
951.7	18∶1/18∶2/22∶6	Na	Oocyte stages 1–2

The underlined molecules were identified from MS/MS analysis by observing the peaks corresponding to the neutral loss of fatty acid moieties. The molecules for which partial fragment ions could be obtained were identified by an online database search (http://www.hmdb.ca/labm/jsp/mlims/MSDbParent.jsp) and Simlipid software.

### Identification of lipid species by MS/MS analysis

**Figure 5:**
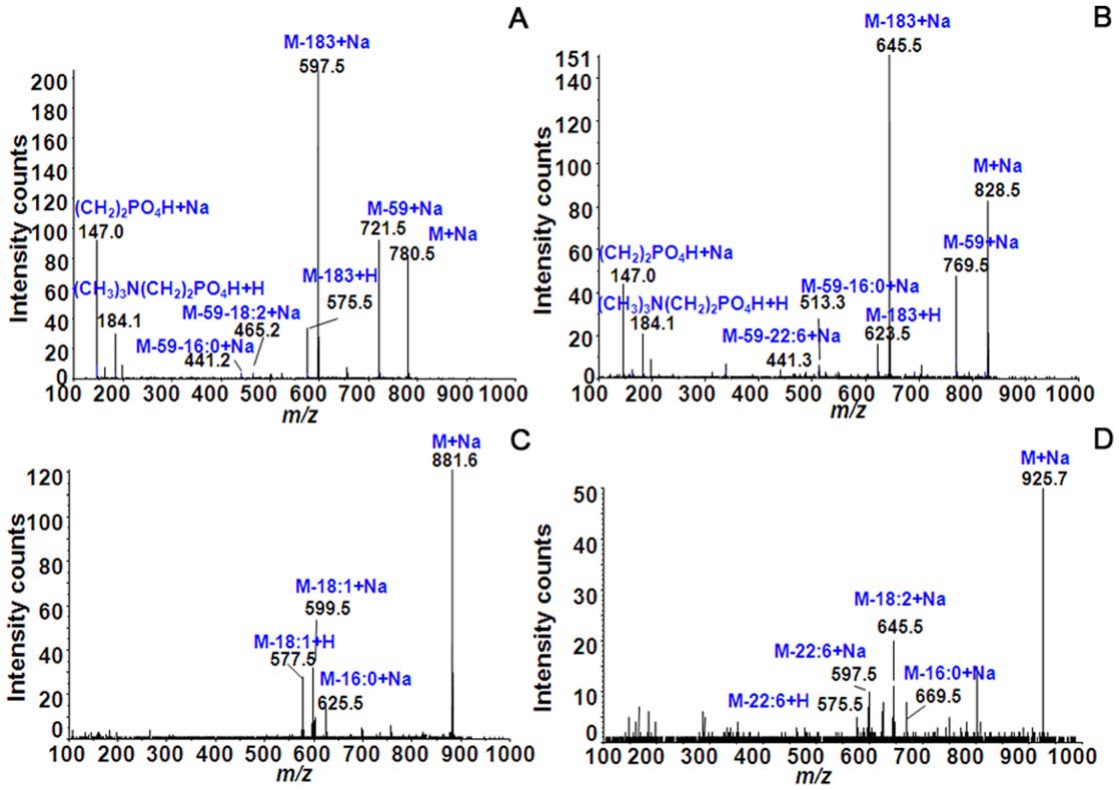
The identification of PCs (A,B) and TAG (C,D) species by MS/MS analysis. Molecules found from the TLC-blot and IMS analyses were subjected to identification by MS/MS analysis. In case of the PCs, the example molecules *m/z* 780.5 (A) and *m/z* 828.5 (B) are shown as fragmented ions after MS/MS analysis. These molecules were characterized as PC (16∶0/18∶2)+Na and (16∶0/22∶6)+Na, respectively. The identification of these molecules was based on the observed fragmented ions that showed the neutral loss property of FA moieties. In the case of TAGs, the example molecules *m/z* 881.6 (C) and 925.7 (D) are shown. In the TAG mass spectra, the peaks that correspond to neutral loss of FA could be observed directly. The molecule at *m/z* 881.6 was assigned as TAG (16∶0/18∶1/18∶1)+Na, and the molecule at *m/z* 925.7 was assigned as TAG (16∶0/18∶2/22∶6)+Na according to the observed neutral loss peaks. The molecular species of each molecule are shown in Tables 2 (PCs) and 3 (TAGs).

The identities of the lipid species were verified by MS/MS analysis. In case of the PCs, the molecules at *m/z* 780.5 (Fig. 5A) and *m/z* 828.5 (Fig. 5B) showed fragmented ions after MS/MS analysis. These molecules were identified as PCs 16∶0/18∶2 with [M+Na]^+^ and 16∶0/22∶6 with [M+Na]^+^. The identification of these molecules was based on the observed fragmented ions that showed neutral loss of the FA moieties constituting the PC molecules. Based on previous studies [26], [27], the molecule at *m/z* 780.5 showed a fragmented major peak at *m/z* 146.9, which represented a neutral loss of the PC head group adducted with Na^+^ and corresponded to [(CH_2_)_2_ PO_4_H+Na]^+^. Moreover, we were able to detect two minor peaks at *m/z* 441.2 and 465.2, which corresponded to neutral losses of trimethylamine ((CH_3_)_3_N) from the PC head group plus FAs (59+280 and 59+256). The molecular weights of 256 and 280 Da could be assigned as FAs 16∶0/18∶2. This identification was also matched with an online database search (http://www.hmdb.ca/labm/jsp/mlims/MSDbParent.jsp) and Simlipid software version 1.0, which helped to increase the confidence of the lipid identification. Similarly, the molecule at *m/z* 828.5 showed neutral losses of FAs by the presence of 2 peaks at *m/z* 441.2 and *m/z* 513.2, corresponding to trimethylamine ((CH_3_)_3_N) from the head group plus FAs (59+328 and 59+256). The identification of the other lipid molecules was performed in a similar manner; and the identified lipid species are shown in Table 2 for the PCs. In case of the TAGs, the molecules at *m/z* 881.6 (Fig. 5C) and 925.7 (Fig. 5D) are shown as fragmented ions after MS/MS analysis. The identification of TAGs is different from that of PCs. According to a previous study [27], the peak corresponding to the neutral loss of a FA can be observed directly in the mass spectrum. For the molecule at *m/z* 881.6, the peaks at *m/z* 625.5 and 599.5 were neutral losses of 16∶0 and 18∶1, and *m/z* 577.5 was adducted with H^+^ after replacing the Na^+^ of *m/z* 599.5. Therefore, this molecule could be assigned as TAG 16∶0/18∶1/18∶1 with [M+Na]^+^. In addition, the molecule at *m/z* 925.7 was assigned as TAG 16∶0/18∶2/22∶6 with [M+Na]^+^, since the observed peaks *m/z* 669.5, 645.5, and 597.5 corresponded to neutral losses of 16∶0, 18∶2, and 22∶6, respectively. Each molecular species is shown in Table 3 for TAG identification. The underlined molecules represent those that were observed as fragmented ions from MS/MS, whereas the non-underlined molecules could be obtained as partially fragmented ions. These partially fragmented ions were further verified to validate their identities by an online database search and SimLipid software.

## Discussion

As shown in our TLC analysis (Fig. 2), the increasing PC and TAG intensities during ovarian maturation in *P. merguiensis* were similar to those of another penaeid shrimp, *P. semisulcatus*. The major FAs in the mature ovaries of a penaeid shrimp, *L. vannamei*, were reported to be 16∶0, 16∶1, 18∶0, 18∶1, 20∶4, 20∶5, and 22∶6 [5] and in *P .monodon* were 16∶0, 16∶1, 18∶0, 18∶1, 18∶2, 20∶4, 20∶5, and 22∶6 [28]. In the present study, the major FAs detected in the mature ovaries of *P. merguiensis* comprised 16∶0, 16∶1, 18∶0, 18∶1, 18∶2, 20∶4, 20∶5, and 22∶6, while the minor FAs comprised 14∶0, 15∶0, 17∶0, 20∶1, and 20∶2 (Fig. 3). Thus, the composition of the major FAs in *P. merguiensis* ovary detected in this study are quite similar to those reported in *L. vannamei* [5] and *P. monodon* [28]. However some FAs were found to be different to those reported in *M. rosenbergii* [8] and *L. Vannamei*. In *P. Merguiensis* and *L. Vannamei* the proportion of FAs 16∶0 and 18∶0 found to be decreased whereas in *M. rosenbergii* the first FA found to be increased and the later FA remained unchanged. In addition proportion of FAs 22∶6 found to be decreased in *M. rosenbergii* and *L. Vannamei* but not in *P. Merguiensis*. The different proportion of some FAs among shrimp species indicating that the requirement to synthesis and store each FA to utilize during ovarian maturation in each shrimp species is different. Therefore to promote the successive ovarian maturation to the specific shrimp species the knowledge of FA requirement is important as to be used to formulate the suitable feeding diet. Moreover, because of the sensitivity of the technique used in our study, we were also able to detect the minor FAs comprising 14∶0, 15∶0, 17∶0, and 20∶1.

Based on our findings, the changing FA levels in relationship to the total lipids during the ovarian maturation cycle exhibited 3 patterns: decreasing, increasing, and unchanging (Fig. 3B). In the first group FAs comprised 16∶0, 18∶0, 20∶4, and 20∶5. We observed high levels of the saturated FAs 16∶0 and 18∶0 in the stage I ovary as well as in early stage oocytes (Oc 1, 2). In a previous study, FAs 16∶0 and 18∶0 were found to be the major saturated FAs in mammals and fishes [29]. Further, these FAs were suggested to be nutritional sources and to serve as precursors for the syntheses of other FAs. In *M. rosenbergii*, relatively high levels of these FAs are present in the early stages of ovarian development, indicating they may be required for the development of the earlier oocyte stages, as they could be effectively digested in this prawn [30]. Furthermore, the prawns were also able to convert these saturated FAs to monounsaturated FAs to be stored in the ovary and subsequently utilized by the embryo [30]. Therefore, the decreased proportional levels of these FAs as the ovary becomes more mature may be due to their increased utilization to satisfy the energy requirement of the developing oocytes, and also due to their conversion to unsaturated lipids. Moreover, we found that the relative amounts of FAs 20∶4 and 20∶5 also decreased (Fig. 3B) as in *P. monodon* [11]. During the progression of the ovarian cycle, the reproductive hormone prostaglandin is required to enhance ovarian maturation and ovulation [11]. Therefore, the reduced levels of 20∶4 and 20∶5 in *P. merguiensis* may be due to the high requirement of prostaglandin biosynthesis, which uses these FAs as precursors. This is supported by the same study showing that high prostaglandin in the feed shortened the period of gonadal maturation in this shrimp [11].

In the second group, the FAs with increased levels during ovarian maturation comprised 14∶0, 16∶1, 18∶2, 18∶1, 20∶1, and 22∶6. In 2 studies of *P. monodon*, increasing levels of 18∶2 and 22∶6 [28] and 16∶1, 18∶1, and 22∶6 [12] were found to be important for the reproductive performance of the shrimp, i.e., the fecundity and percentage of hatching. Similarly, the present study detected increased relative amounts of 16∶1, 18∶1, 18∶2, and 22∶6 during the ovarian cycle, implying that these FAs are essential for oocyte and embryo development. Even though the FAs 14∶0 and 20∶1 belonged to the minor group, they also significantly increased during ovarian maturation. Thus, they may also have essential roles in oocyte development.

In the last group, the FAs displaying unchanged levels comprised 15∶0, 17∶0, and 20∶2. We classified the level of FA 17∶0 as an unchanged level, since first we observed slightly decreased proportion of FA 17∶0 from ovaries stage I to III and finally the level of this FA at stage IV remained stable. This finding agree with the previous study in *L. vannamei* which found the level of FA 17∶0 slightly fluctuated [5]. All FAs in this group are minor components, indicating that they might not be directly involved in reproductive performance.

In addition to examining the fatty acid compositions and their changing levels in all 4 ovarian stages, the present study is the first one to report on the spatial distribution of lipids in various oocyte stages during ovarian maturation in a penaeid shrimp (Fig. 4, S1, S2). Normally, the lipid composition is reported as total FAs extracted from a tissue that usually includes FAs from PCs and TAGs [3], [5], [6], [7], [13], [14], [31]; however, the specific localization of each lipid has never been investigated. In this study, we were able to detect the distribution of PCs, TAGs, and associated FAs directly in the ovarian tissue sections by IMS and thereby provide information on the accumulation of these lipids during oocyte maturation, and enabling us to identify the FAs that might be required for each stage of oocyte development. Previous study in penaeid shrimp has shown that during sexual maturation, the remarkable increases of PCs and TAGs were observed [12]. A study in *Penaeus japonicus* [32] showed that the ovarian maturation period was lengthened when the diet did not contain phospholipids. Hence PC may be essential for oocyte maturation. As for TAG, this molecule was incorporated into the eggs and act as energy source to supply embryogenesis, hatching, egg quality and nauplii development [12]. Furthermore in our present study, by using IMS technique, we could localize and classify PC- and TAG-associated FAs that were selectively stored in each oocyte stages during ovarian maturation (Table 2, 3 and Fig. S1, S2). PC and TAG accumulation exhibited 3 different patterns. PCs and TAGs which were found to be specifically localized in early stage of oocytes, Oc 1,2, comprising PC 16∶0/16∶1 and TAG 18∶1/18∶2/22∶6. This suggests that these molecules are essential only for early stages of oocytes, but not required for late stage of oocytes, Oc 3,4. The second group was the PC and TAG molecules that were present in all stages of oocytes, comprising of PC 16∶1/20∶4, 16∶0/22∶6, 18∶3/22∶6, 18∶1/22∶6, 20∶5/22∶6, 22∶6/22∶6 and TAG 16∶0/16∶1/18∶3, 16∶0/18∶1/18∶3, 16∶0/18∶1/18∶1, 16∶0/18∶2/22∶6. This suggests that these molecules play important roles in maintaining and stimulating oocyte development, since we could observe them being maintained at the same intensity in all oocyte stages. The last group was PCs and TAGs that were observed to be highly intense in Oc 3,4 comprising of PC 16∶0/18∶2, 16∶0/18∶1, 18∶2/18∶2, 18∶1/18∶2, 18∶0/18∶2, 16∶0/20∶4, 18∶2/20∶5, 18∶1/20∶4, 18∶0/20∶4, 16∶0/22∶6, 18∶2/22∶6, 18∶1/22∶6 and TAG 16∶1/16∶1/16∶1, 16∶0/16∶1/16∶1, 16∶0/16∶1/20∶5, 16∶0/16∶0/20∶5, 16∶0/16∶0/20∶4, 18∶1/16∶0/20∶5. We suggest that these molecules might act as the essential nutritional and energy source for developing embryo as they were accumulated in the late and mature stages of oocytes.

The reproductive capacity of fish and shrimp depends on many factors, such as hormones [33], dietary intake [34], and the amounts and types of lipids in the diet [35]. Amongst lipids it has been reported that polyunsaturated (PUFA) and highly unsaturated fatty acids (HUFA) play the key roles in stimulating female fecundity. In many species of fish it has been clearly demonstrated that HUFA (DHA, 22∶6; EPA, 20∶5 and ARA, 20∶4) are essential for female reproduction and fecundity, as well as the quantity and quality of the offspring, even-though the exact mechanism and targets of each fatty acid are not known [9], [28], [34], [35]. Furthermore, in *M. rosenbergii* feeding female broodstocks with high level of 18∶2n-6 together with n-3 HUFA increased fecundity, egg hatchability and larval quality [30]. It is known that some PUFA, for examples, linolenic acid (LNA), 18∶3n-3, and linoleic acid LA, 18∶2n-6, can serve as precursors for EPA/DHA and ARA, respectively [30]. Taken together the high level of PUFA in *P. merguiensis* ovary indicates that this is an essential group of fatty acids that must be included in the broodstock feed. It is notable that LNA is lacking in this prawn, hence inclusion of EPA and DHA in the feed is also essential. Whereas the detection of high level of saturated FAs 16∶0 and 18∶0 suggested that these molecules could serve as major energy source for the maturing oocytes. Thus, information on lipid compositions and localization, as performed in this study, could provide basic knowledge on FA requirements for oocyte differentiation during ovarian maturation in this shrimp species, and could be used in formulating feed for female broodstocks to enhance their fecundity and larval production.

## Materials and Methods

### Histology of the developing ovary

Thirteen female *P. merguiensis* shrimp (weighing between 18–22 g, with length between 20–25 cm.) were obtained for each of the 4 stages of the ovarian cycle from the Coastal Aquatic Feed Research Institute, Petchburi Province, Thailand. Shrimp ovaries were quickly dissected and immediately fixed in Bouin's fixative overnight. The fixed samples were processed for paraffin embedding by using a LEICA TP 120 tissue processor (Leica, Germany). The samples were finally embedded in paraffin blocks and cut by a rotary microtome (Leica RM2235, Germany) into5-µm thick sections, which were mounted on glass slides and stained with H&E. The H&E images of the ovaries were captured by a Nikon ECLIPSE E 600 light microscope equipped with an E66 digital camera (Nikon, Tokyo, Japan).

### Identification of lipids by thin-layer chromatography

#### Extraction of lipids

The extraction procedure was performed as follows: ovarian samples were weighed, homogenized, and extracted with 0.1 g/ml of extraction solution (chloroform∶methanol 2∶1, v/v). The homogenates were placed in a glass tubes (pre-treated with chloroform∶methanol 1∶1, v/v), and sonicated more than 10 times by a Microson Ultrasonic Cell Disruptor XL-2000 (Wakenyaku Co. Ltd., Kyoto, Japan) for a duration of 10 sec per sonication, with 5-sec stops. The sonicated samples were incubated overnight at room temperature. The samples were centrifuged at 3,000×*g*, and the supernatants were collected into new glass tubes. Afterwards, the glass tubes were tightly wrapped with parafilm and stored at −80°C until sample analysis.

### 2.2 Thin layer chromatography

The separation procedure followed a previously described method [36], [37], [38]. In brief, 3 µl of the extracted lipids were applied to a 10-×10-cm high performance thin layer chromatography (HPTLC) plate (Merck, Darmstadt, **Germany**) and then completely dried before being placed into the TLC chamber. To enable better separation and prevent lipid diffusion, the applied areas were prevented from being immersed in the separation buffer. For PC separation, the HPTLC plate was incubated in a separation buffer composed of methylacetate∶n-propanol∶chloroform∶0.25% KCl at the ratio 25∶25∶10∶9 (v/v/v/v), whereas for TAG separation the plate was incubated in a buffer composed of N-hexane∶diethylether∶acetic acid at the ratio 80∶30∶1 (v/v/v), for 30 min. After separation, the PC and TG plates were completely dried. To observe the separated lipids, primuline solution (1 mg of primuline in 100 ml of 80% acetone in water) was sprayed onto the HPTLC plates, which were then completely dried, and the bands were visualized by UV light (FAS-III; **Toyobo** Co. Ltd., Osaka, Japan). The intensities of the lipid bands in each ovarian stage were analyzed by ImageJ software version 1.41 and expressed as means with standard deviations. Significant differences among the ovarian stages were statistically tested by using Scheffe's method, with *p*<0.05.

### Analyses of fatty acid composition by gas chromatography-mass spectrometry

#### Methylation and purification of FAs

The extracted FAs from each ovarian stage were subjected to methylation using an acid methylation **kit (NacalaiTesque**, **Inc**., Kyoto, **Japan**) before being analyzed by gas chromatography-mass spectrometry (GC-MS). The internal standard arachidicacid (20∶0) was added to the extracts at concentration 0.4 µg/µl. The extracts were then completely dried by a **TurboVap LV** Evaporation System (Caliper Life Sciences, Hopkinton, MA, USA) and continuously aerated with nitrogen gas to prevent the oxidative reaction. After the methylation procedure, the samples were purified by using a fatty acid methyl ester purification kit (Nacalai Tesque, Inc.), according to the manufacture's instruction. The purified FAs were stored at −80°C until analyzed.

#### Fatty acid identification by gas chromatography-mass spectrometry

To identify and analyze FA compositions, 1 µl of the purified samples of each ovarian stage were injected into a GC-MS QP-2010 Plus (Shimadzu corp. Kyoto, Japan) system equipped with a DB-5MS column (30×0.25 mm I.D., 0.25 µm; D.F., Agilent technologies). The column temperature was held at 210°C. The column pressure was increased from 110 kPa to 380 kPa at a rate of 7 kPa/min (from 110 kPa to 180 kPa) and 10 kPa/min (from 180 kPa to 380 kPa). Significant differences (*p*<0.05) among the lipids of the ovarian stages were tested using Scheffe's method.

### Localization of lipids by imaging mass spectrometry

The fresh frozen ovaries from each stage were serially cut at −20°C by a CM 1950 cryostat (Leica Microsystems) at a thickness of 10 µm, and the sections mounted onto indium tin oxide (ITO)-coated microscope glass slides (BrukerDaltonics, Bremen, Germany). The ovarian tissues were not embedded in any embedding medium lest any residual polymers on the tissue sections might interfere and degrade the mass spectra [39]. The tissues sections were stored at −80°C until being analysed. Before applied matrix, the tissue sections were completely dried by a hair dryer. The matrix, 2,5-dihydroxybenzoic acid (DHB) (Bruker Daltonics), was dissolved at 50 mg/ml in 70% methanol and 0.1% TFA, and then sprayed onto the tissue sections by using a 0.2-mm nozzle caliber airbrush (Procon Boy FWA Platinum, Tokyo, Japan). The sprayed samples were analysed using an Ultraflex II MALDI TOF/TOF mass spectrometer (Bruker Daltonics) in positive ion mode. The mass spectra were acquired in the mass ranges between *m/z* 500–1,500, and the laser frequency was optimized to 200 Hz. The number of laser irradiations was 200 shots in each spot. For obtaining a clear signal distribution on the tissue sections, the resolution of the images was optimized by setting the laser raster width at 35 µm per laser spot. After analysis, the images were constructed by using the imaging software tool, Flex imaging 2.1 (Bruker Daltonics). The H&E stained was performed by using the same tissue sections after IMS analysed (Fig. 4A).

### Identification of lipid species by MS/MS analysis

The extracted lipids were subjected to tandem mass spectrometry (MS/MS) analysis. To validate the identity of each lipid species, the QSTAR Elite high-performance, hybrid quadrupole Time-of-Flight mass spectrometer (Applied Biosystems, Foster city, CA) was used to acquire fragment ion spectra. The collision energy was optimized and adjusted between 40 to 50 V during fragmentation of the lipid molecules. An online lipid database search, using Metabolite MS Search (http://www.hmdb.ca/labm/jsp/mlims/MSDbParent.jsp) and the software SimLipid Version 1.0 (PREMIER Biosoft International, Palo Alto, CA, USA), was performed to identify molecules from their partial fragment spectra.

## Supporting Information

Figure S1 **Imaging mass spectrometry showing the distribution of each PC molecule in various ovarian stages.** The distributions of these molecules exhibit 3 patterns: the first group is mainly distributed in Oc 1,2; the second group, Oc 3,4; the last group, all oocytes. The molecular species and their distributions are shown in Table 2.
(TIF)

Figure S2 **Imaging mass spectrometry showing the distribution of TAG molecules in various ovarian stages.** Similar to the PCs, the TAGs also show 3 patterns of distribution, i.e., mainly in Oc1,2, in oocyte 3,4, or in all stages. The molecular species and their distributions are shown in Table 2.
(TIF)
