# Supplementary figures and images for: Composition and Localization of Lipids in Penaeus merguiensis Ovaries during the Ovarian Maturation Cycle as Revealed by Imaging Mass Spectrometry
Source: PLoS One. 2012 Mar 14;7(3):e33154. doi: 10.1371/journal.pone.0033154 (PMC3303810; doi:10.1371/journal.pone.0033154)

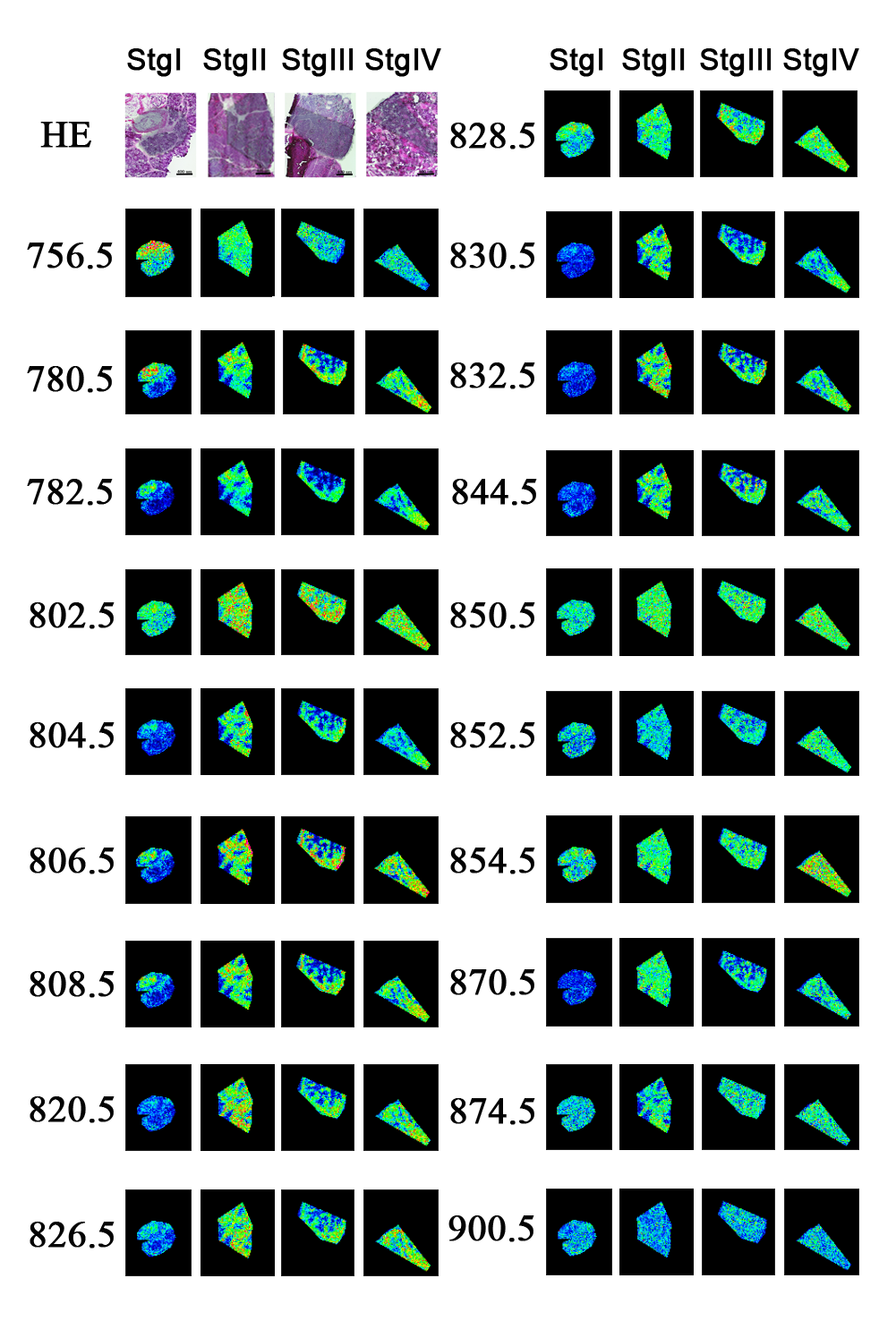

Supplement: Figure S1 — Imaging mass spectrometry showing the distribution of each PC molecule in various ovarian stages. The distributions of these molecules exhibit 3 patterns: the first group is mainly distributed in Oc 1,2; the second group, Oc 3,4; the last group, all oocytes. The molecular species and their distributions are shown in Table 2. (TIF) [file pone.0033154.s001.tif]

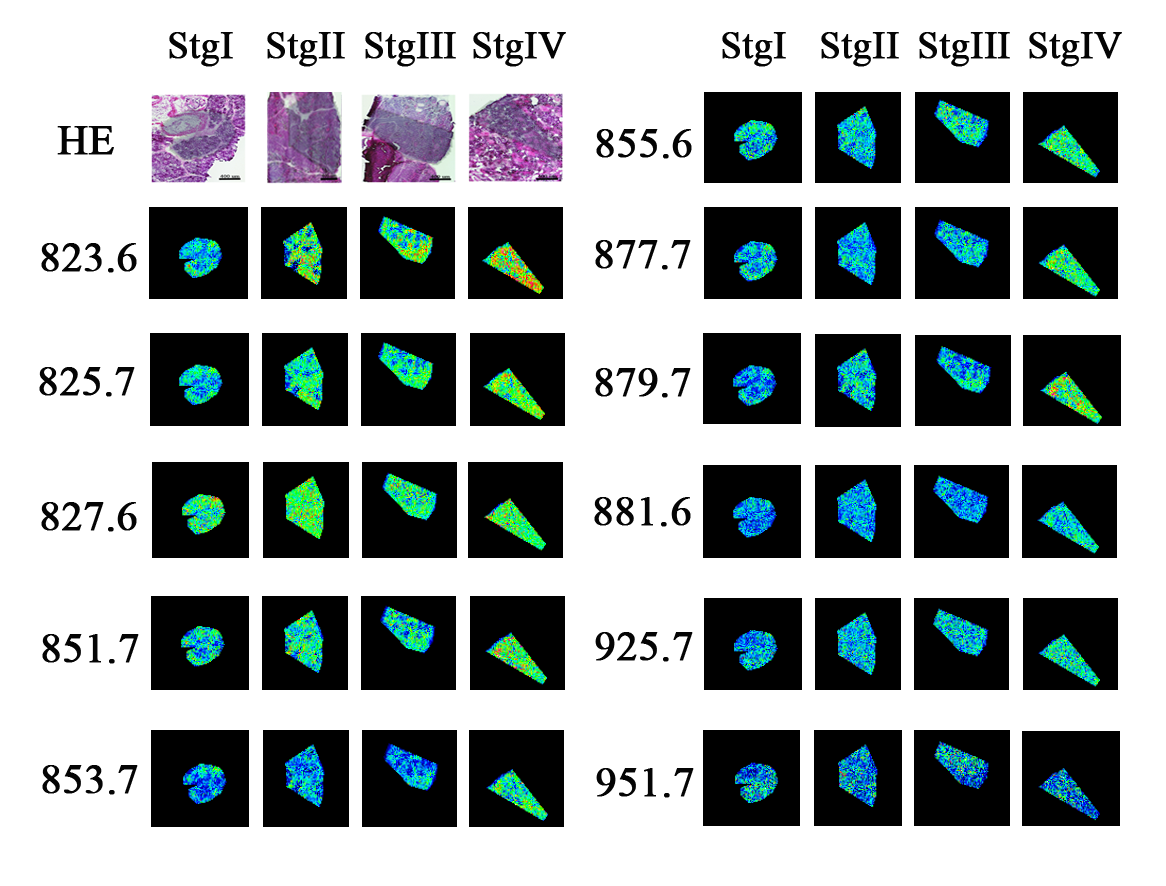

Supplement: Figure S2 — Imaging mass spectrometry showing the distribution of TAG molecules in various ovarian stages. Similar to the PCs, the TAGs also show 3 patterns of distribution, i.e., mainly in Oc1,2, in oocyte 3,4, or in all stages. The molecular species and their distributions are shown in Table 2. (TIF) [file pone.0033154.s002.tif]
